# The Medical Record Visiting List

**Published:** 1884-11

**Authors:** 


THE MEDICAL RECORD VISITING LIST, OR PHYSICIAN'S DIARY. Wm. Wood & Co., New York.

This handsome and well arranged Visiting List for 1885 has already made its appearance. The following is the table of contents:

The Metric System—Thermometric Scales—Table of Signs—Almanac—Table for Estimating the Probable Duration of Pregnancy —Approximate Equivalents of Small Weights—Doses of Drugs used for Subcutaneous Injection—Doses of Common and Rare Drugs —Drugs Suited for Atomization, Inhalation, Doses, etc.—Disinfectants—The Urine; Amount, Color, Odor, Chemical Analysis—Poisons and their Antidotes—Emergencies—Facts—Lister's Antiseptic Solution—Treatment of Asphyxia from Various Causes—Visiting List with Special Memoranda—Consultation Practice—Obstetric Engagements—Record of Obstetrical Practice—Record of Vaccination—Register of Deaths—Nurses' Addresses—Addresses of Patients and others—Cash Account.

A good visiting list is indispensable to a busy practitioner, and we know of no one that we can more confidently recommend for its true merit than this.

